# Medium‐chain fatty acid receptor GPR84 deficiency leads to metabolic homeostasis dysfunction in mice fed high‐fat diet

**DOI:** 10.1096/fba.2024-00075

**Published:** 2024-10-17

**Authors:** Akari Nishida, Ryuji Ohue‐Kitano, Yuki Masujima, Hazuki Nonaka, Miki Igarashi, Takako Ikeda, Ikuo Kimura

**Affiliations:** ^1^ Department of Molecular Endocrinology, Graduate School of Pharmaceutical Sciences Kyoto University Kyoto Japan; ^2^ Department of Biological & Environmental Chemistry Kindai University Iizuka Japan; ^3^ Laboratory of Molecular Neurobiology, Graduate School of Biostudies Kyoto University Kyoto Japan; ^4^ Department of Applied Biological Science, Graduate School of Agriculture Tokyo University of Agriculture and Technology Fuchu Tokyo Japan

**Keywords:** decanoates, fatty acids, GPR84, high‐fat diet, obesity, type 2 diabetes

## Abstract

Overconsumption of food, especially dietary fat, leads to metabolic disorders such as obesity and type 2 diabetes. Long‐chain fatty acids, such as palmitoleate are recognized as the risk factors for these disorders owing to their high‐energy content and lipotoxicity. In contrast, medium‐chain fatty acids (MCFAs) metabolic benefits; however, their underlying molecular mechanisms remain unclear. GPR84 is an MCFA receptor, particularly for C10:0. Although evidence from in vitro experiments and oral administration of C10:0 in mice suggests that GPR84 is related to the metabolic benefits of MCFAs via glucose metabolism, its precise roles in vivo remain unclear. Therefore, the present study investigated whether GPR84 affects glucose metabolism and metabolic function using *Gpr84*‐deficient mice. Although *Gpr84*‐deficient mice were lean and had increased endogenous MCFAs under high‐fat diet feeding conditions, they exhibited hyperglycemia and hyperlipidemia along with lower plasma insulin and glucagon‐like peptide‐1 (GLP‐1) levels compared with wild‐type mice. Medium‐chain triglyceride (C10:0) intake suppressed obesity, and improved plasma glucose and lipid levels, and increased plasma GLP‐1 levels in wild‐type mice; however, these effects were partially attenuated in *Gpr84*‐deficient mice. Our results indicate that long‐term MCFA‐mediated GPR84 activation improves the dysfunction of glucose and lipid homeostasis. Our findings may be instrumental for future studies on drug development with GPR84 as a potential target, thereby offering new avenues for the treatment of metabolic disorders like obesity and type 2 diabetes.

## Graphical abstract

Evidence suggests that the MCFA receptor GPR84 is linked to the metabolic benefits of medium‐chain fatty acids (MCFAs). However, its specific roles in vivo remain unclear. Although Gpr84‐deficient mice were lean and had increased endogenous MCFAs under high‐fat diet feeding conditions, they exhibited hyperglycemia and hyperlipidemia along with lower plasma insulin and glucagon‐like peptide‐1 levels compared with wild‐type mice. These findings indicate that long‐term MCFA‐mediated GPR84 activation may improves the dysfunction of glucose and lipid homeostasis.

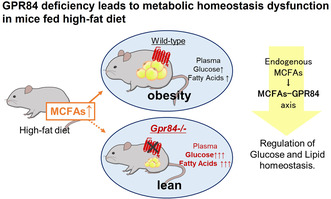

**Abbreviations**
BAT: brown adipose tissue
CCK: cholecystokinin
ELISA: enzyme‐linked immunosorbent assay
GIP: gastric inhibitory polypeptide
GLP‐1: glucagon‐like peptide‐1
HFD: high‐fat diet
LC‐MS/MS: liquid chromatography with tandem mass spectrometry
MCFA: medium‐chain fatty acid
MCT: medium‐chain triglyceride
NC: normal chow
NEFA: non‐esterified fatty acid; SEM, standard error of the mean
UPLC: ultra performance liquid chromatography
WAT: white adipose tissue

## 1 INTRODUCTION

While diet plays a crucial role in daily nutrition, overconsumption of food can lead to metabolic disorders such as obesity, type 2 diabetes, hepatic steatosis, cardiovascular disease, and related conditions. Obesity, a serious health crisis worldwide, is associated with increased mortality and contributes to a cluster of symptoms collectively referred to as metabolic syndrome, which includes hyperglycemia, dyslipidemia, hypertension, weight gain, and insulin resistance.^1^, ^2^ In recent decades, it has become evident that all macronutrients, including lipids, carbohydrates, and proteins, play important roles in regulating inflammatory responses and energy metabolism. Obesity results from long‐term imbalance between energy intake and expenditure, and affects the effector pathways of several hormones and metabolites.^3^ The risk factors for obesity include excessive food intake and physical inactivity, as well as genetic susceptibility.

Medium‐chain triglycerides (MCTs) comprise medium‐chain fatty acids (MCFAs), represent a unique form of dietary fat, contain approximately 10% fewer calories than other factors for metabolic disorders due to their high energy content and lipotoxicity. MCFAs offer various health benefits and aid in weight loss.^4^, ^5^, ^6^, ^7^ Shorter chain MCFAs are easily absorbed in the intestine due to their higher water solubility compared with long‐chain fatty acids. MCFAs are also readily metabolized as a fuel source since they are transported directly to the liver.^8^ This improved metabolic conversion ensures that the calories in MCFAs are effectively turned into immediate fuel for muscles and organs, rather than stored as fat.^9^

Free fatty acids act as energy sources and also influence physiological functions including hormone secretion, neurotransmission, and immune response via free fatty acid‐specific receptors, such as GPR40 and GPR120 for long‐chain fatty acids and GPR41 and GPR43 for short‐chain fatty acids.^4^, ^10^, ^11^, ^12^, ^13^, ^14^ MCFAs also bind to a specific receptor, GPR84,^4^, ^15^, ^16^ which is expressed predominantly in the bone marrow, immune cells, lungs, and metabolically related tissues and is coupled to the pertussis toxin‐sensitive Gi/o protein.^15^, ^16^, ^17^ Although several studies using *Gpr84*‐deficient mice have demonstrated that GPR84 plays an important role in metabolic and immune responses,^18^, ^19^, ^20^, ^21^ comprehensive and integrated data are lacking and the molecular mechanisms underlying these processes remain unclear.

We recently reported that the dietary MCT (TriC10) regulates glucose metabolism via GPR84‐mediated glucagon‐like peptide‐1 (GLP‐1) secretion^18^ and that endogenous MCFAs are increased in the plasma through hepatic lipid metabolism under high‐fat diet (HFD) feeding^19^ in mice. These findings suggest that the MCFA receptor GPR84 is involved in glucose homeostasis and metabolic functions under Western diet feeding conditions. Therefore, in the present study, we aimed to determine whether GPR84 affects glucose metabolism and metabolic function using an HFD‐induced obese *Gpr84*‐deficient mouse model.

## 2 MATERIALS AND METHODS

### 2.1 Animals and diet

Male C57BL/6J mice were purchased from Japan SLC (RRID: IMSR_JAX: 000664, Shizuoka, Japan). *Gpr84* ^*−/−*^ mice (C57BL/6J background) were generated using the CRISPR/Cas9 system as previously described.^19^ The mice were housed in a conventional animal room at 24°C under a 12 h light/dark cycle, and acclimated to the CLEA Rodent Diet (CE‐2; CLEA Japan, Inc., Tokyo, Japan) for 1 week prior to treatment. For short‐term treatment, 7‐week‐old mice were fed normal chow (NC) or an HFD with 60% kcal fat (D12492, Research Diets Inc., New Brunswick, NJ, USA) for 5 weeks. For long‐term treatment, 4‐week‐old mice were fed an HFD for 12 weeks. The diet composition is shown in the (Table S1). For MCT diet trial, mice were fed an MCT diet for 12 weeks at 4 weeks of age. Diets were formulated as either lard or MCT (decanoate [C10:0] triglyceride: Tricaprin; TCI., Tokyo, Japan). The diet compositions are shown in (Table S2). Body weight was measured once a week during the experiment. Food intake was measured manually and presented as daily average intake. All the mice were sacrificed using deep isoflurane‐induced anesthesia, and the blood and tissues were collected. The soleus muscle of the femur was collected. All experimental procedures involving mice were performed in accordance with the guidelines of the Committee on the Ethics of Animal Experiments of the Kyoto University Animal Experimentation Committee (Lif‐K23012), and all efforts were taken to minimize suffering.

### 2.2 Biochemical analyses

Plasma non‐esterified fatty acid (NEFA) (LabAssay™ NEFA; FUJIFILM Wako Pure Chemical Corporation, Osaka, Japan), plasma triglyceride (LabAssay™ Triglyceride; FUJIFILM Wako Pure Chemical Corporation), and plasma total cholesterol (LabAssay™ Cholesterol; FUJIFILM Wako Pure Chemical Corporation) levels in mice were measured according to the manufacturer's instructions. Blood glucose levels were measured using a handheld glucometer (OneTouch® Ultra®; LifeScan, Milpitas, CA, USA). Plasma insulin (Insulin enzyme‐linked immunosorbent assay [ELISA] kit [RTU]; Shibayagi, Gunma, Japan), plasma cholecystokinin (cholecystokinin enzyme immune assay [EIA] Kit, Merck Millipore, Darmstadt, Germany), plasma gastric inhibitory polypeptide (Rat/Mouse GIP [total] ELISA, Merck Millipore), and plasma GLP‐1 (glucagon‐like peptide‐1 [Active] ELISA kit; Merck Millipore) levels were measured using ELISA according to the manufacturer's instructions. To measure plasma GLP‐1 levels, samples were treated with a dipeptidyl peptidase‐IV inhibitor (Merck Millipore), which prevents the degradation of active GLP‐1.

### 2.3 FA measurement

Plasma FA measurement was conducted following a previously described method with modifications.^19^ Briefly, the plasma samples were first mixed with methanol containing an internal standard (C17: 1) and then mixed with chloroform and 0.5 M potassium chloride for lipid extraction. The samples were centrifuged at 500×*g* at 17°C for 10 min, and the lower layer containing FAs was collected and dried. Subsequently, the samples were resuspended in chloroform: methanol (1: 3, v/v) and subjected to liquid chromatography with tandem mass spectrometry (LC–MS/MS) analysis using an ultra‐performance LC system (UPLC, Waters, Milford, MA, USA) equipped with an Acquity UPLC system coupled to a Waters Xevo TQD mass spectrometer (Waters). Samples were separated using a methanol gradient in 10 mM ammonium formate aqueous solution on an ACQUITY UPLC BEH C18 column (2.1 × 150 mm, 1.7 μm; Waters).

### 2.4 Glucose tolerance tests

To assess their glucose tolerance, mice were administered glucose (2 g/kg body weight) by oral gavage following a 16‐h fast. For the insulin tolerance test, mice were fasted for 3 h and then injected with insulin intraperitoneally (0.75 mU/g; Sigma‐Aldrich, St. Louis, MO, USA). Blood glucose concentrations were monitored before injection and at 15, 30, 60, 90, and 120 min postinjection.

### 2.5 RNA isolation and quantitative reverse transcriptase (qRT)‐PCR

Total RNA was extracted from the animal tissues using the RNAiso Plus reagent (TAKARA, Shiga, Japan). Complementary DNA (cDNA) was synthesized from the RNA templates using Moloney murine leukemia virus reverse transcriptase (Invitrogen). qRT‐PCR was performed using the StepOne Real‐Time PCR System (Applied Biosystems) with SYBR Premix Ex Taq II (TAKARA). The PCR conditions were as follows: 95°C for 30 s, followed by 40 cycles of 95°C for 5 s, 58°C for 30 s, and 72°C for 1 min. The mRNA levels of the target genes were calculated using the 2^−ΔΔCt^ method following normalization to the 18S rRNA as the housekeeping gene. The primer sequences used are as follows: 18S, 5′‐CTCAACACGGGAAACCTCAC‐3′ (forward) and 5′‐AGACAAATCGCTCCACCAAC‐3′ (reverse); Tnf, 5′‐GGCAGGTCTACTTTGGAGTC‐3′ (forward) and 5′‐TCGAGGCTCCAGTGAATTCG‐3′ (reverse); Adgre1, 5′‐GATGTGGAGGATGGGAGATG‐3′ (forward) and 5′‐ACAGCAGGAAGGTGGCTATG‐3′ (reverse); Pnpla2, 5′‐CGCAATCTCTACCGCCTCTC‐3′ (forward) and 5′‐ATCCTCCTCTCCAGCCCTCT‐3′ (reverse); Adrb3, 5′‐GGCCCTCTCTAGTTCCCAG‐3′ (forward) and 5′‐TAGCCATCAAACCTGTTGAGC‐3′ (reverse); Acaca, 5′‐CTTCCTGACAAACGAGTCTGG‐3′ (forward) and 5′‐CTGCCGAAACATCTCTGGGA‐3′ (reverse); Srebf1, 5′‐GGAGCCATGGATTGCACATT‐3′ (forward) and 5′‐GGCCCGGGAAGTCACTGT‐3′ (reverse); Pparg, 5′‐TCAGCTCTGTGGACCTCTCC‐3′ (forward) and 5′‐ACCCTTGCATCCTTCACAAG‐3′ (reverse).

### 2.6 RNA sequencing

RNA was extracted from the epididymal adipose tissue of 12‐week‐HFD‐fed mice using the RNAiso Plus reagent (TAKARA) and purified using the RNeasy mini kit (Qiagen, Hilden, Germany). cDNA libraries for RNA sequencing were generated using the NEBNext® Ultra™ II Directional RNA Library Prep Kit (Illumina) and NEBNext Multiplex Oligos for Illumina (Dual Index Primers Set 1), and subsequently sequenced on the Illumina NovaSeq 6000. Each sample yielded approximately 4 gigabases of paired‐end reads with a length of 150 bp. The RNA sequencing data were pre‐processed using trimmomatic‐0.39 to remove adapters and poor‐quality reads.^22^ The quality of the trimmed sequences was assessed using FastQC,^23^ while the reads were aligned to the mouse reference genome (NCBI GRCm39) using STAR.^24^ The raw read counts were normalized using relative log expression and differentially expressed genes (DEGs) were identified across all comparisons. The data were expressed as fold change using the RSEM (version1.3.3) and edgeR.^25^ DEGs were determined based on the following two criteria: false discovery rate (FDR)‐adjusted *p* < 0.05 (the Benjamini‐Hochberg procedure) and |log2 (fold change)| > 0.5. A Gene Set Enrichment Analysis was performed using the Kyoto Encyclopedia of Genes and Genomes (KEGG) database (http://www.genome.jp/kegg/).

### 2.7 Histological analysis

The fresh frozen epididymal adipose tissue was sliced into 12‐μm thick sections using a cryo‐microtome (Leica, Wetzlar, Germany). Immunohistochemical analysis was performed using antibodies against F4/80 (1:1000; Abcam, ab6640) and αSMA (1:300; Cell Signaling Technology, 19245), and the nuclei were stained with DAPI (1:5000; Roche, 10236276001), as described previously.^19^ The frozen sections were fixed with 4% paraformaldehyde, permeabilized with 0.2% Triton X‐100 (Sigma), and blocked with 1% bovine serum albumin (BSA) in PBS. Next, the sections were incubated with primary antibodies, followed by incubation with secondary antibodies conjugated with a fluorescent marker. For hematoxylin–eosin (HE) staining, frozen sections fixed with 4% paraformaldehyde were stained with hematoxylin solution, followed by immersion in 70% ethanol for fixation. Subsequently, the sections were stained with eosin solution for 4 min. After rinsed with running water, the sections underwent dehydration with 70% ethanol, then with 100% ethanol, followed by transparency with xylene. All sections were observed under a microscope (BZ‐X710, Keyence Co., Osaka, Japan).

### 2.8 Statistical analysis

All data are presented as the mean ± standard error of the mean (SEM). GraphPad Prism (GraphPad Software Inc., La Jolla, CA, USA, RRID: SCR_002798) was used for the statistical analyses. Data normality was assessed using the Shapiro–Wilk test. For the statistical comparisons, the Student's *t*‐test (two‐tailed), the Mann–Whitney *U* test (two‐tailed), Tukey–Kramer test, or two‐way ANOVA with the Bonferroni was applied depending on data normality. Statistical significance was set at *p* < 0.05.

## 3 RESULTS

### 3.1 GPR84 deficiency suppresses weight gain in HFD‐fed mice

**FIGURE 1:**
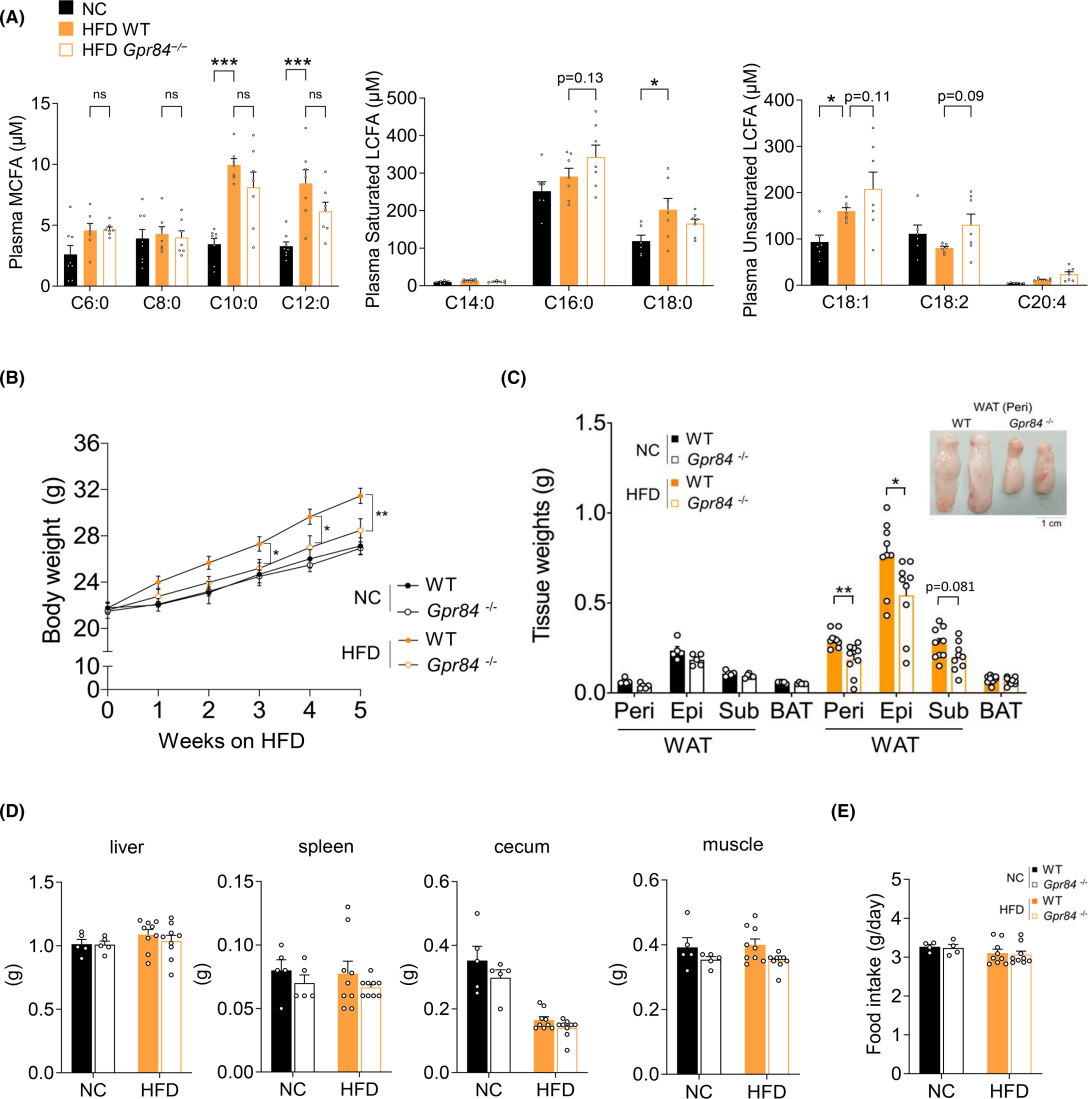
*Gpr84* ^*−/−*^ mice show reduced weight gain under HFD feeding. (A) Plasma MCFA and LCFA contents determined by LC/MS in wild‐type mice (WT) and *Gpr84* ^*−/−*^ mice fed with NC or HFD for 5 weeks (NC‐fed group, *n* = 8; HFD‐fed group, *n* = 7; independent experiments). (B) Body weight changes in WT and *Gpr84* ^*−/−*^ mice fed with NC or HFD, and (C) Adipose tissue weight after 5 weeks (NC‐fed group, *n* = 5 from 8 litters; HFD‐fed group, *n* = 9 from 9 litters; independent experiments). Epi, epididymal adipose tissues; peri, perirenal adipose tissues; sub, subcutaneous adipose tissues; WAT, white adipose tissue; BAT, brown adipose tissue. (D) Tissue weight after 5 weeks (NC‐fed group, *n* = 5 from 8 litters; HFD‐fed group, *n* = 9 from 9 litters; independent experiments). (E) Food intake (NC‐fed group, *n* = 4 from 8 litters; HFD‐fed group, *n* = 9 from 9 litters; independent experiments). ***p < 0.001; **p < 0.01; *p < 0.05 (Tukey–Kramer test (A) Mann–Whitney *U* test (C, D, E) two‐way ANOVA with the Bonferroni (B). All data are presented as the mean ± SEM.

Previously, we found that endogenous MCFAs sufficiently increase in the liver and plasma of wild‐type mice fed an HFD for 5 weeks.^19^ Therefore, in this study, we first confirmed the increase in endogenous MCFAs in plasma of wild‐type mice fed NC or HFD for 5 weeks (Table S1). Compared with NC‐fed mice, HFD‐fed mice had substantially increased plasma endogenous MCFAs, particularly C10:0, which may serve as a potential ligand for the MCFA receptor GPR84 (Figure 1A). Therefore, we exposed *Gpr84* ^*−/−*^ mice to the regimen to investigate the role of GPR84 in the metabolic system. Following HFD feeding, although plasma MCFAs levels were comparable between wild‐type and *Gpr84* ^*−/−*^ mice, plasma long‐chain fatty acids were higher in *Gpr84* ^*−/−*^ than wild‐type mice (Figure 1A). Although the body weights of wild‐type and *Gpr84* ^*−/−*^ mice were comparable under NC feeding conditions, the body weights of HFD‐fed *Gpr84* ^*−/−*^ mice were significantly lower than those of HFD‐fed wild‐type mice during growth (Figure 1B). Further, the weight of white adipose tissue (WAT) was comparable between wild‐type and *Gpr84* ^*−/−*^ mice fed NC for 5 weeks; however, the weight of WAT, but not of brown adipose tissue (BAT), was significantly lower in *Gpr84* ^*−/−*^mice than in wild‐type mice that were fed HFD for 5 weeks (Figure 1C), whereas the weights of other tissues were comparable between wild‐type and *Gpr84* ^*−/−*^ mice (Figure 1D). Additionally, the food intake was also similar between the two groups (Figure 1E). Thus, GPR84 deficiency confers a remarkable resistance to HFD‐induced weight gain.

### 3.2 *Gpr84*‐deficient mice exhibit dysfunctions of metabolic homeostasis under HFD feeding

**FIGURE 2:**
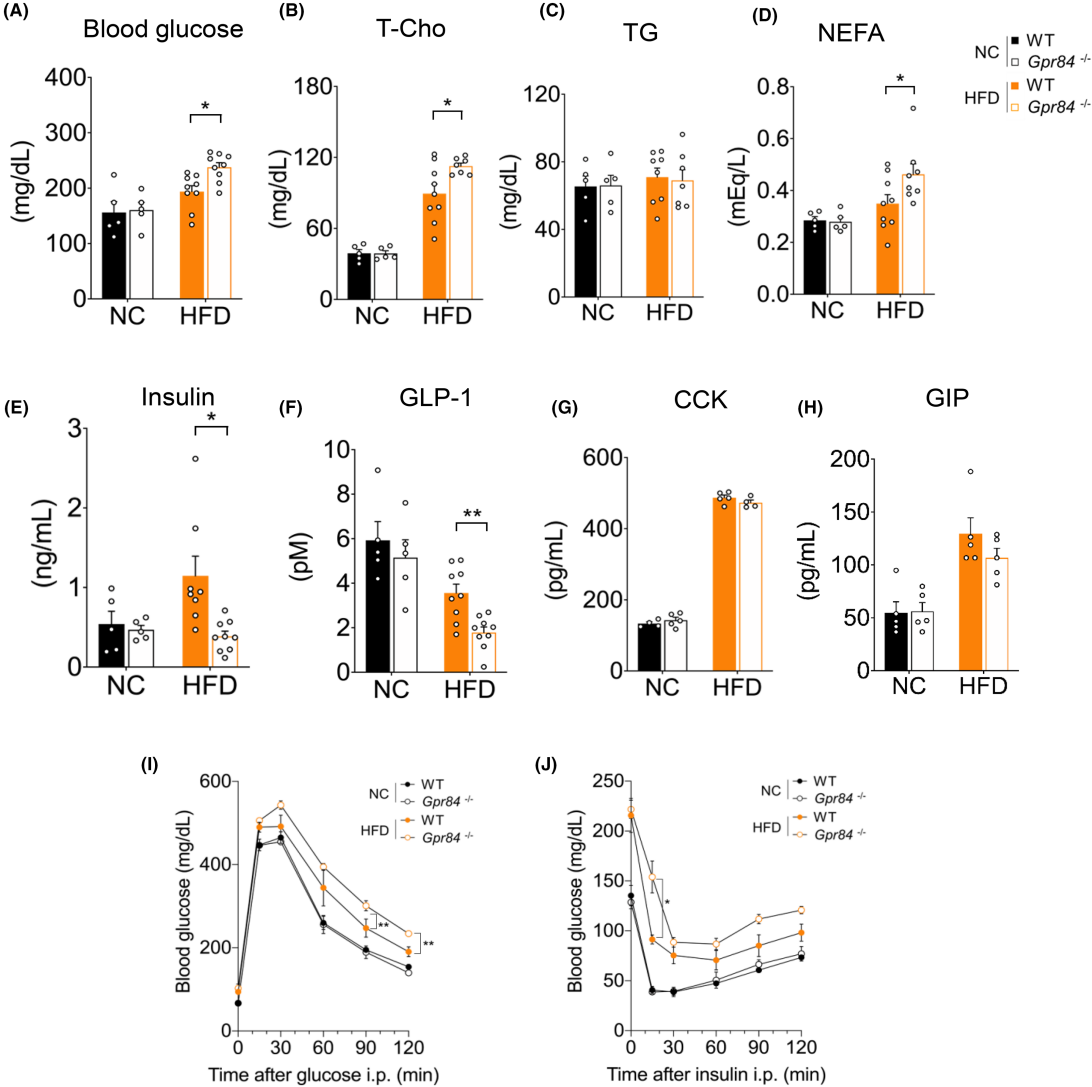
*Gpr84* ^*−/−*^ mice show dysfunction of glucose homeostasis under HFD feeding. (A) Blood glucose, (B) plasma total cholesterol (T‐Cho), (C) plasma triglycerides (TGs), and (D) plasma non‐esterified fatty acids (NEFAs) (NC‐fed group, *n* = 5 from 8 litters; HFD‐fed group, *n* = 7–9 from 9 litters; independent experiments). (E) Plasma insulin levels, and (F) Plasma GLP‐1 levels in WT and *Gpr84* ^*−/−*^ mice following NC or HFD feeding for 5 weeks (NC‐fed group, *n* = 5; HFD‐fed group, *n* = 8–9; independent experiments). (G) Plasma CCK levels and (H) Plasma GIP levels in WT and *Gpr84* ^*−/−*^ mice following NC or HFD feeding for 5 weeks (NC‐fed group, *n* = 4–5; HFD‐fed group, *n* = 4–5; independent experiments). NC fed group from 8 litters; HFD fed group from 9 litters. (I) Glucose tolerance test (GTT) and (J) insulin tolerance test (ITT) (NC‐fed group, *n* = 5; HFD‐fed group, *n* = 6). **p < 0.01; *p < 0.05 (Mann–Whitney *U* test (A, E, H) Student's *t* test (B–D, F, G) two‐way ANOVA with the Bonferroni (I, J). All data are presented as the means ± SEM.

Although *Gpr84* ^*−/−*^ mice showed resistance to weight gain under HFD feeding for 5 weeks, the plasma glucose levels of 5‐week HFD‐fed *Gpr84* ^*−/−*^ mice were significantly higher than those of 5‐week HFD‐fed wild‐type mice; however, there were no significant differences between *Gpr84* ^*−/−*^ and wild‐type mice fed NC for 5 weeks (Figure 2A). Similarly, although the plasma total cholesterol levels in 5‐week NC‐fed *Gpr84* ^*−/−*^ mice were comparable to those of 5‐week NC‐fed wild‐type mice, the plasma cholesterol levels of 5‐week HFD‐fed *Gpr84* ^*−/−*^ mice were significantly higher than those of 5‐week HFD‐fed wild‐type mice (Figure 2B). The plasma triglyceride levels, on the other hand, were comparable among the four groups (Figure 2C). Additionally, while the plasma NEFA levels were not significantly different between wild‐type and *Gpr84* ^*−/−*^ mice under NC diet, the plasma NEFA levels of 5‐week HFD‐fed *Gpr84* ^*−/−*^ mice were significantly higher than that of 5‐week HFD‐fed wild‐type mice (Figure 2D).

Hyperglycemia in lean individuals is generally due to insufficient insulin secretion as well as insulin resistance.^26^ Therefore, we next investigated glucose tolerance in *Gpr84* ^*−/−*^ mice fed with HFD for 5 weeks. The plasma insulin levels were comparable between wild‐type and *Gpr84* ^*−/−*^ mice fed with NC; however, they were significantly lower in HFD‐fed *Gpr84* ^*−/−*^ mice than in HFD‐fed wild‐type mice (Figure 2E). GPR84 activation by C10:0 has been reported to promote the secretion of the incretin hormone GLP‐1 in mouse enteroendocrine STC‐1 cells and MCT oral administration experiments in mice.^18^ As expected, the plasma GLP‐1 levels were not significantly different between wild‐type and *Gpr84* ^*−/−*^ mice fed the NC diet; however, the plasma GLP‐1 levels were significantly lower in HFD‐fed *Gpr84* ^*−/−*^ mice than in HFD‐fed wild‐type mice (Figure 2F). Additionally, the levels of other gut hormones^27^ in the plasma, such as cholecystokinin (CCK) and gastric inhibitory polypeptide (GIP), were substantially increased by HFD feeding; however, the levels were not significantly different between wild‐type and *Gpr84* ^*−/−*^ mice under both NC and HFD feeding conditions (Figure 2G,H). HFD‐induced glucose intolerance and insulin resistance were significantly exacerbated in HFD‐fed *Gpr84* ^*−/−*^ mice compared with wild‐type mice (Figure 2I,J). Therefore, although GPR84 deficiency suppressed HFD‐induced weight gain, it aggravated the HFD‐induced increases in plasma glucose and lipid levels, thereby impairing glucose homeostasis and hypoinsulinemia under HFD feeding.

### 3.3 *Gpr84*‐deficient mice exhibit severe metabolic dysfunction under long‐term HFD feeding

**FIGURE 3:**
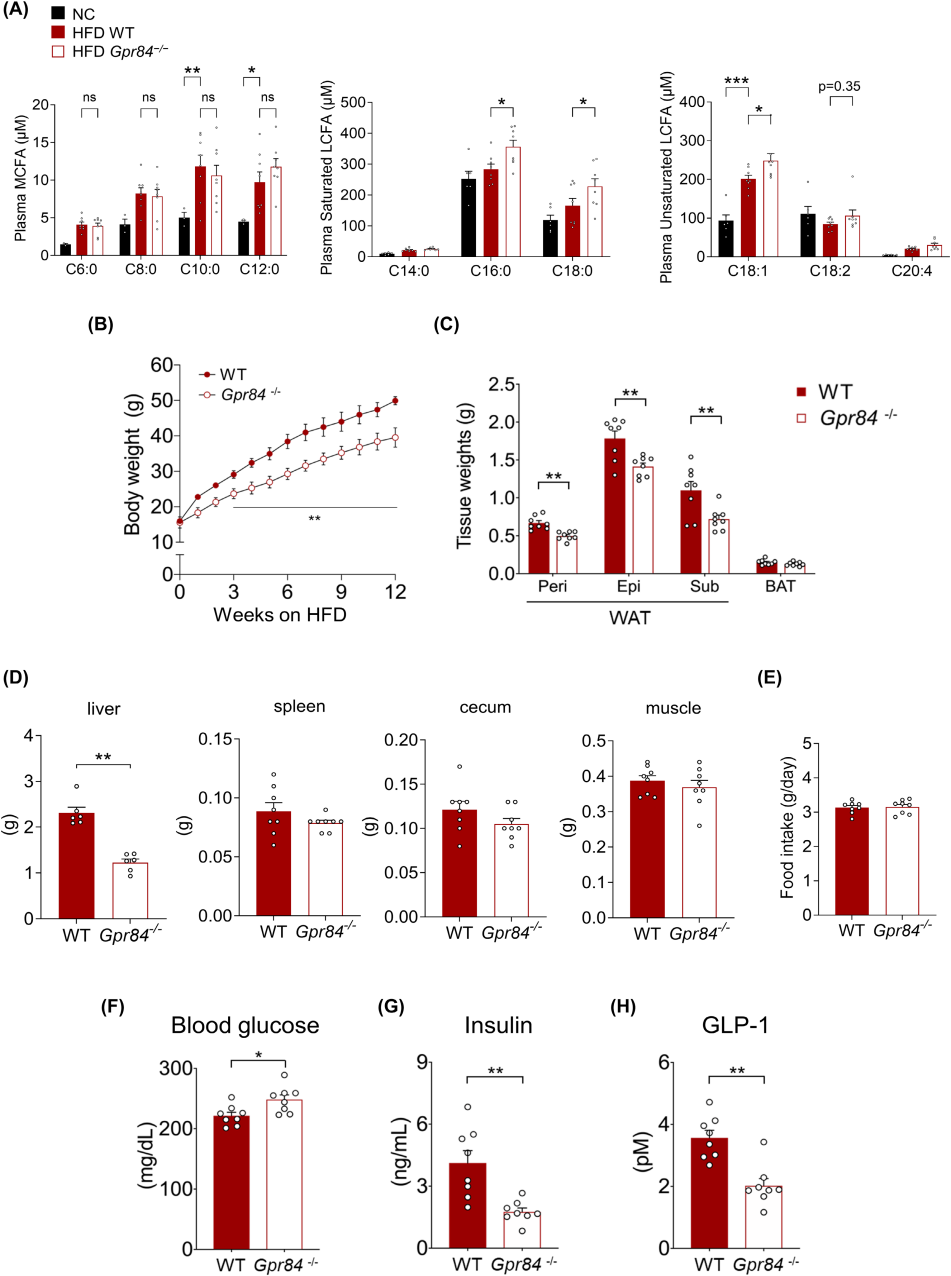
*Gpr84* ^*−/−*^ mice show severe dysfunction of glucose homeostasis under long‐term HFD feeding. (A) Plasma MCFA and LCFA contents determined by LC/MS in WT mice fed on HFD for 12 weeks (NC‐fed group, *n* = 3–6; HFD‐fed group, *n* = 8; independent experiments). (B) Body weight changes in WT and *Gpr84* ^*−/−*^ mice during HFD feeding and (C) Adipose tissue weight after 12 weeks (*n* = 7–8 from 9 litters; independent experiments). Epi, epididymal; peri, perirenal; sub, subcutaneous; BAT, brown adipose tissue; WAT, white adipose tissue. (D) Tissue weight after 12 weeks (*n* = 6–8 from 9 litters; independent experiments). (E) Food intake (*n* = 8 from 9 litters; independent experiments). (F) Blood glucose level in WT and *Gpr84* ^*−/−*^ mice after HFD intervention for 12 weeks (*n* = 8). (G) Plasma insulin and (H) plasma GLP‐1 levels (*n* = 8 from 9 litters; independent experiments). ***p < 0.001; **p < 0.01; *p < 0.05 (Tukey–Kramer test (A) two‐way ANOVA with the Bonferroni (B) Student's *t* test (C–H). All data are presented as the mean ± SEM.

Long‐term HFD feeding leads to type 2 diabetes symptoms including obesity, hyperglycemia, hyperlipidemia, and impaired insulin sensitivity in mice.^11^ Therefore, we investigated how long‐term HFD feeding influences metabolic changes in *Gpr84* ^*−/−*^ mice by providing HFD to 4‐week‐old mice for 12 weeks.^11^ Compared with 12 weeks of NC feeding, 12 weeks of HFD feeding considerably increased plasma endogenous MCFAs in mice, especially C10:0 (Figure 3A). Subsequently, we investigated the role of GPR84 in the metabolic system using *Gpr84* ^*−/−*^ mice. Under 12 weeks HFD feeding, although plasma MCFAs levels were comparable between wild‐type and *Gpr84* ^*−/−*^ mice, plasma long‐chain fatty acids were higher in *Gpr84* ^*−/−*^ than wild‐type mice (Figure 3A). The body weights of HFD‐fed *Gpr84* ^*−/−*^ mice were markedly lower than those of wild‐type mice during growth (Figure 3B). The weight of WAT, but not BAT, was also significantly lower in HFD‐fed *Gpr84* ^*−/−*^ mice compared with wild‐type mice (Figure 3C), whereas the weights of other tissues, beside the liver, 19 were comparable between wild‐type and *Gpr84* ^*−/−*^ mice (Figure 3D). Additionally, food intake was similar (Figure 3E). Although the plasma glucose levels of both groups of HFD‐fed mice indicated hyperglycemia, the plasma glucose levels of HFD‐fed *Gpr84* ^*−/−*^ mice were higher than those of HFD‐fed wild‐type mice (Figure 3F). Additionally, the plasma insulin levels were significantly lower in HFD‐fed *Gpr84* ^*−/−*^ mice than in HFD‐fed wild‐type mice (Figure 3G). Furthermore, plasma GLP‐1 levels were significantly lower in HFD‐fed *Gpr84* ^*−/−*^ mice compared with wild‐type mice (Figure 3H). Thus, GPR84 deficiency exacerbates glucose metabolism dysfunction induced by long‐term HFD feeding.

### 3.4 *Gpr84*‐deficient mice exhibit lipid metabolic dysfunction but not inflammation in WAT under HFD feeding

**FIGURE 4:**
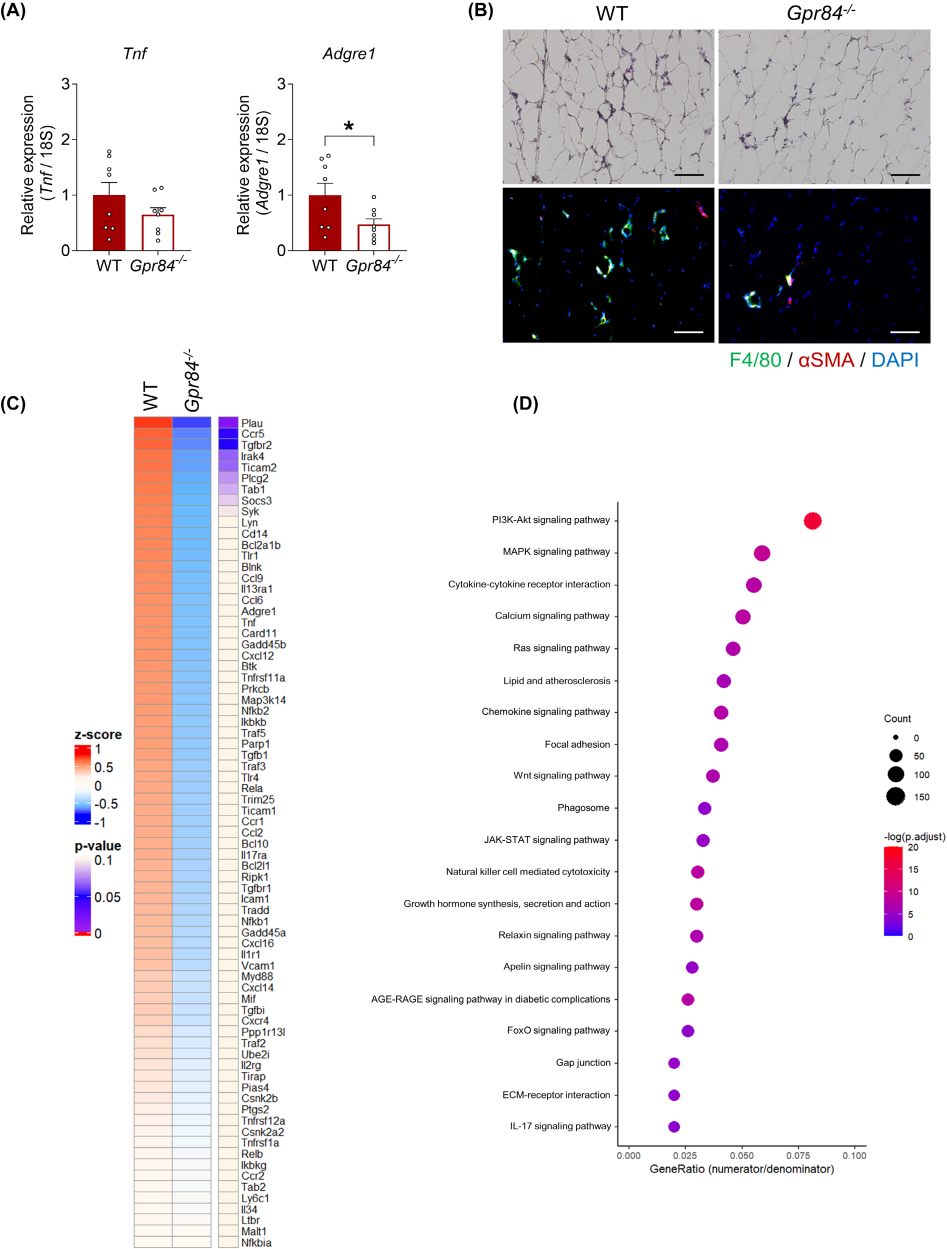
*Gpr84* ^*−/−*^ mice show improvement in inflammatory response under long‐term HFD feeding. (A) *Tnf* and *Adgre1* mRNA expression in WAT (*n* = 8). (B) Inflammation level in WAT. Scale bar = 100 μm. (C) RNA‐Seq transcriptome profiling in WAT of WT and *Gpr84* ^*−/−*^ mice fed the HFD for 12 weeks. Heatmap shows the results of two‐dimensional hierarchical clustering of 75 genes associated with inflammation (*n* = 4 per group). (D) Top 20 enriched Kyoto Encyclopedia of Genes and Genomes (KEGG) pathways of differentially expressed genes (DEGs). *p*‐values were adjusted for multiple testing using Bonferroni correction. *p < 0.05 (Student's *t*‐test: A). All data are presented as the mean ± SEM.

We investigated the crucial tissue for metabolic dysfunctions of HFD‐fed *Gpr84* ^*−/−*^ mice. GPR84 is expressed in immune cells and involved in inflammatory responses.^15^ Therefore, we assessed the inflammation in WAT of HFD‐fed *Gpr84* ^*−/−*^ mice. Unexpectedly, the expression of the inflammatory marker gene *Tnf* (tumor necrosis factor α) and macrophage marker gene *Adgre1* significantly decreased in WAT of HFD‐fed *Gpr84* ^*−/−*^ mice compared with that in wild‐type mice (Figure 4A). Similarly, F4/80‐positive macrophages were decreased in HFD‐fed *Gpr84* ^*−/−*^ mice compared with that in HFD‐fed wild‐type mice (Figure 4B). Moreover, we conducted RNA sequencing and KEGG pathway analyses to compare the WAT of HFD‐fed wild‐type and *Gpr84* ^*−/−*^ mice and observed a relationship between the chronic inflammation‐related pathways, which were attenuated in the WAT of HFD‐fed *Gpr84* ^*−/−*^ mice (Figure 4C,D). Therefore, HFD‐fed *Gpr84* ^*−/−*^ mice exhibited metabolic benefits, such as improvement of inflammation, as well as anti‐obesity and loss of WAT weights.

**FIGURE 5:**
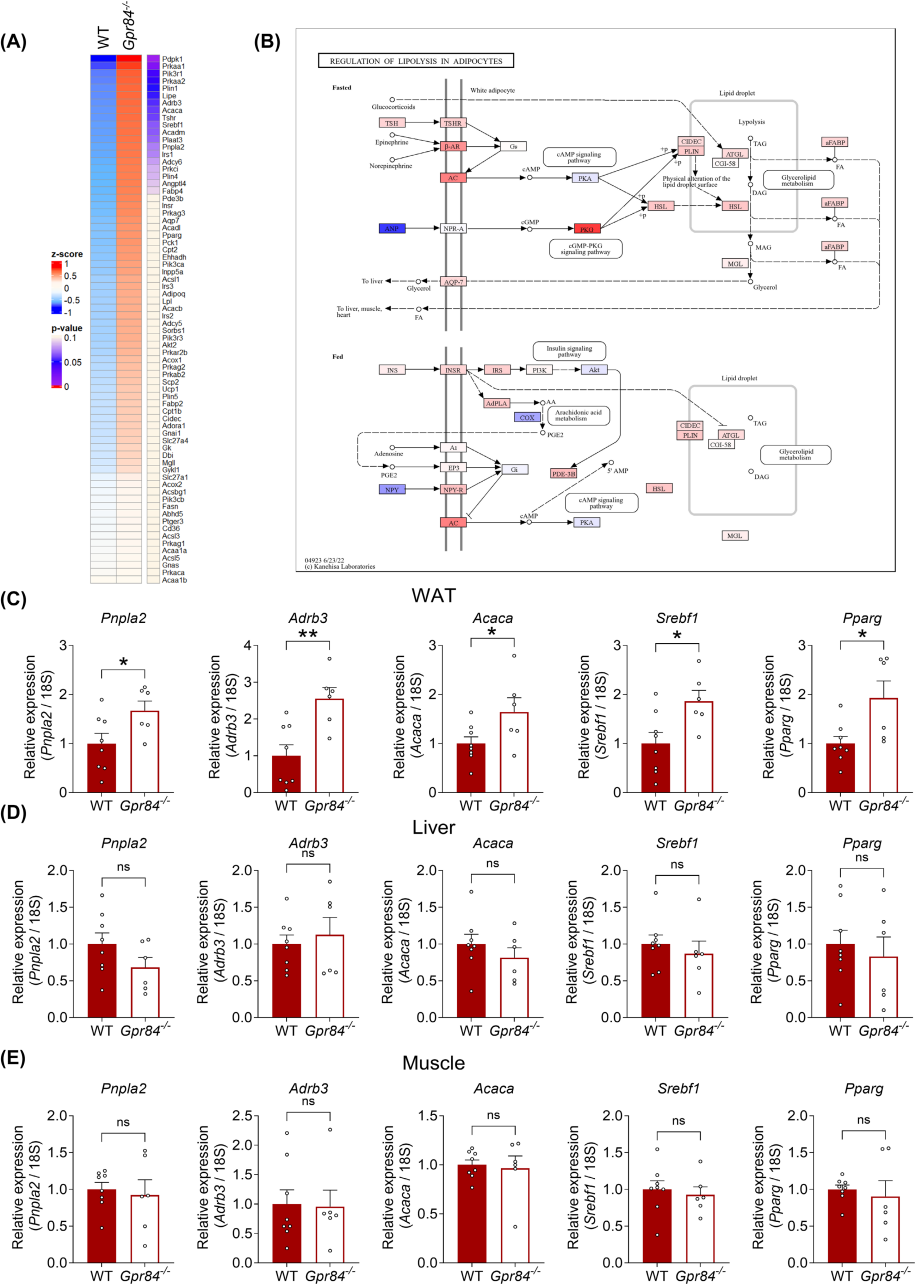
*Gpr84* ^*−/−*^ mice show dysfunctions of lipid metabolism under long‐term HFD feeding. (A) RNA‐Seq transcriptome profiling in WAT of WT and *Gpr84* ^*−/−*^ mice fed the HFD for 12 weeks. Heatmap shows the results of two‐dimensional hierarchical clustering of 72 genes associated with lipid metabolism (*n* = 4 per group). (B) KEGG pathway analysis related to lipid metabolism. (C–E) mRNA expression levels of lipid metabolism‐related genes in WAT (C), liver (D), and muscle (E) measured in WT and *Gpr84* ^*−/−*^ mice during HFD feeding (*n* = 6–8). **p < 0.01, *p < 0.05 (Student's *t* test (C–E). All data are presented as the mean ± SEM.

Moreover, RNA sequencing and KEGG pathway analyses showed a relationship between the lipid metabolic pathways. Lipogenesis‐related genes along with HFD feeding and lipid degradation‐related genes were significantly increased in WAT of HFD‐fed *Gpr84* ^*−/−*^ mice (Figure 5A,B). In the qRT‐PCR experiments, theses lipid metabolism‐related genes in HFD‐fed *Gpr84* ^*−/−*^ mice were significantly increased in WAT but not in the liver or muscle (Figure 5C–E). Therefore, HFD‐fed *Gpr84* ^*−/−*^ mice exhibited increase of expression of lipogenesis‐related genes along with HFD feeding and lipid degradation‐related genes.

### 3.5 GPR84 activation partially mediate the metabolic benefits of MCT intake

**FIGURE 6:**
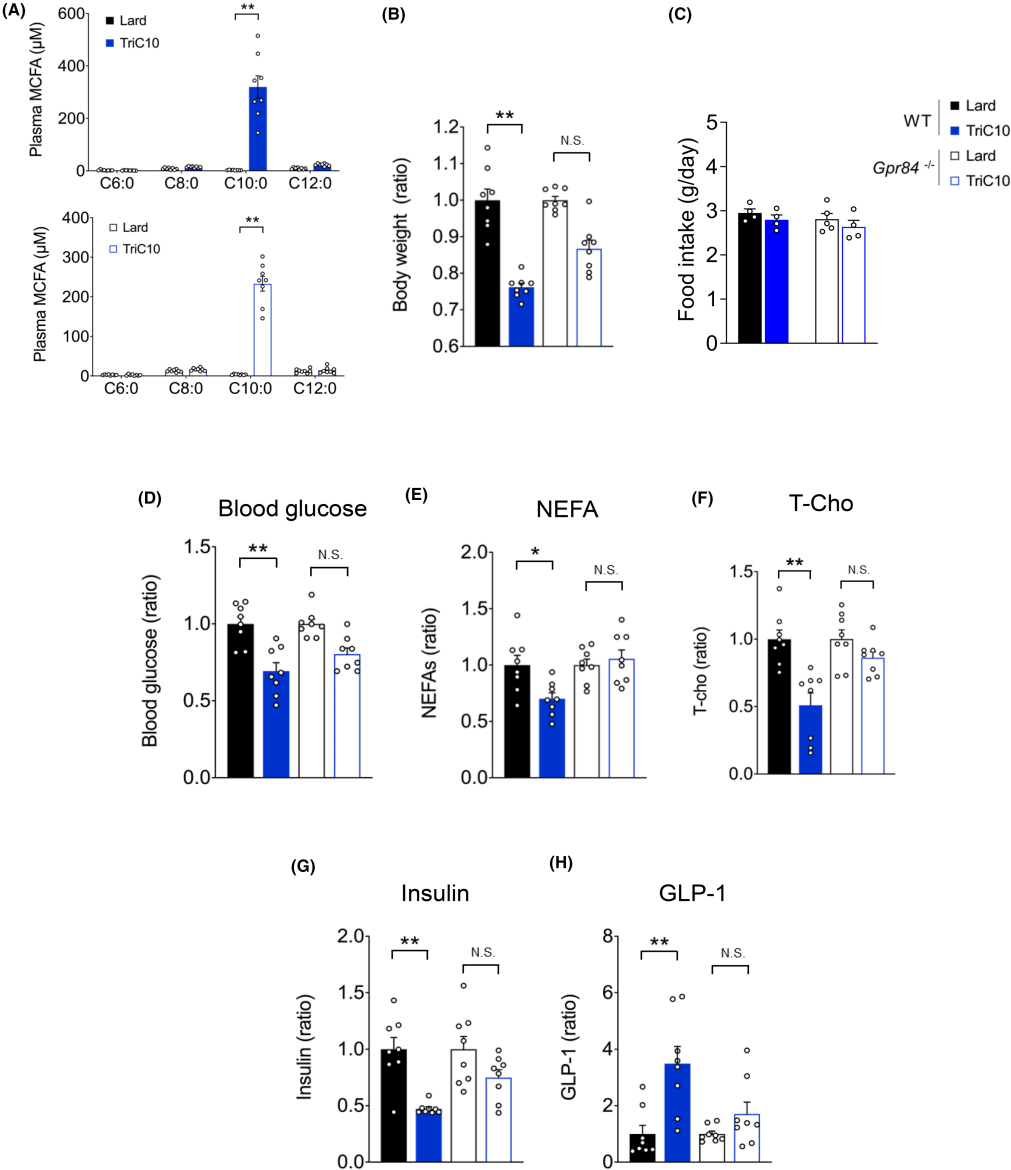
Metabolic benefits of MCT intake are partially mediated by GPR84 activation. (A) MCFA concentration in the plasma of mice fed with TriC10‐supplemented HFD (*n* = 8). (B) Body weight gain for 12 weeks in Lard‐ or MCT diet‐fed mice (*n* = 8). (C) Food intake (*n* = 4–5). (D) Blood glucose levels in Lard or MCT diet‐fed mice at 16 weeks of age were measured after fasting for 5 h (*n* = 8). (E) Non‐esterified fatty acids (NEFAs), and (F) total cholesterol (T‐cho) levels in Lard or MCTs diet‐fed mice. (G) Plasma insulin and (H) plasma GLP‐1 levels (*n* = 8). **p < 0.01; *p < 0.05 (Mann–Whitney *U* test (A, F–H) Student's *t* test (B–E). All data are presented as the means ± SEM.

Finally, we investigated the relationship between metabolic functions via GPR84 and MCT‐induced changes in metabolic parameters after switching to a 20% fat diet in which lard was substituted with MCT (decanoate [C10:0] triglyceride; TriC10). Mice were fed lard or TriC10 for 12 weeks. Plasma C10:0 level was markedly increased in both wild‐type and *Gpr84* ^*−/−*^ mice fed the TriC10 diet compared with mice fed the lard (Figure 6A, Table S2). The body weights of TriC10 diet‐fed mice were significantly lower than those of lard‐fed mice (Figure 6B). The food intake was comparable between the two groups (Figure 6C). In addition, the plasma glucose levels of TriC10 diet‐fed mice were significantly lower than those of lard‐fed mice after 12 weeks (Figure 6D). Plasma NEFA and total cholesterol levels in TriC10 diet‐fed mice were also lower than those in lard diet‐fed mice (Figure 6E, F). Moreover, plasma insulin levels of TriC10 diet‐fed mice were significantly lower than those of lard‐fed mice (Figure 6G). Plasma GLP‐1 levels in TriC10 diet‐fed mice were significantly higher than those in lard diet‐fed mice (Figure 6H). However, all these beneficial effects were found to be attenuated in *Gpr84* ^*−/−*^ mice (Figure 6B,D–H). Thus, MCT (TriC10) intake efficiently prevented obesity, and its effects were partially mediated by GPR84.

## 4 DISCUSSION

While dietary MCTs offer numerous health benefits and the MCFA receptor GPR84 is believed to be related to positive immune and metabolic effects induced by MCT,^18^, ^19^, ^20^, ^21^ the precise molecular mechanism and the relationship of GPR84 with MCTs remain unclear. The present study demonstrated that GPR84 deficiency exacerbates HFD‐induced type 2 diabetes in mice and that the metabolic improvement effects due to MCT intake are partially mediated by GPR84 activation.

In this study, *Gpr84*‐deficient mice exhibited type 2 diabetes symptoms including leanness, hyperglycemia, and hyperlipidemia under HFD feeding conditions, whereas no such metabolic phenotypes were observed in NC‐fed *Gpr84*‐deficient mice. Furthermore, HFD feeding increased plasma MCFA in mice (~20 μM) compared with NC feeding (<5 μM). These indicate that plasma MCFA concentration reaches to sufficient levels for GPR84 activation (EC50: ~8 μM) under HFD feeding, but not NC feeding. In contrast to our results, previous studies reported that *Gpr84*‐deficient mice exhibited only a mild impairment in glucose tolerance as a metabolic phenotype.^20^, ^21^ This difference may have arisen because of the lower fat balance used by these previous studies compared with our diet composition. Further studies are needed to clarify endogenous MCFA production via lipid metabolism under HFD‐fed conditions.

*Gpr84*‐deficient mice fed HFD exhibited metabolic phenotypes such as glucose metabolism dysfunction, along with lower plasma insulin. This may be caused by the reduction of GLP‐1 secretion in the intestine of *Gpr84*‐deficient mice. In support our current results, we previously reported that GPR84 was expressed in the ileum and oral administration of C10:0 improved glucose intolerance by GLP‐1 secretion through intestinal GPR84 activation.^18^ On the other hand, GPR84 is also expressed in WAT and immune cells,^15^, ^16^, ^18^, ^19^ and GPR84 activation in these cells affects metabolic phenotypes.

Our studies showed that inflammation in WAT of *Gpr84*‐deficient mice fed HFD was attenuated compared with wild‐type mice. Meanwhile, metabolic dysfunctions in Gpr84‐deficient mice may cause lipid turnover in WAT of *Gpr84*‐deficient mice fed HFD, since lipogenesis and lipid degradation related genes were elevated in WAT but not in muscle or liver of *Gpr84*‐deficient mice. However, further studies using tissue‐specific *Gpr84‐*deficient mice are required to verify this factor.

The metabolic benefits provided by MCT (TriC10) intake were partially abolished in *Gpr84*‐deficient mice, indicating that not all physiological functions of MCFAs depend on GPR84. For example, besides GPR84, GPR40 also is activated by MCFAs such as C10:0 and C12:0.^4^ This suggests the involvement of multiple receptors in mediating the effects of MCFAs. However, the specific benefits of MCFAs, particularly C10:0, on glucose homeostasis appear to be largely mediated by GPR84. This conclusion is supported by the observation that the beneficial effects on glucose regulation provided by C10:0 intake were significantly reduced in *Gpr84*‐deficient mice. This suppression indicates that GPR84 plays a crucial role in the metabolic improvements associated with C10:0 intake. Therefore, while GPR40, and possibly other receptors, contributed to the physiological effects of MCFAs, GPR84 is important for the enhancement of glucose homeostasis observed following C10:0 consumption. The partial loss of these benefits in *Gpr84*‐deficient mice underscores the significant role of GPR84 in mediating these specific metabolic effects.

As *Gpr84* expression induces various inflammatory responses,^15^, ^28^ GPR84 was initially known as a proinflammatory receptor.^24^ However, recent reports,^19^, ^29^, ^30^ including our previous findings, have confirmed that GPR84 also suppresses inflammation under different conditions, indicating that GPR84 is an inflammatory modulator. In the present study, we demonstrated that GPR84 activation may lead to metabolic benefits in HFD‐induced diabetic mice. Therefore, our findings could pave the way for research on developing drugs or supplements targeting GPR84, with potential applications in treating metabolic disorders such as obesity and type 2 diabetes.

Our results showed that GPR84 deficiency in mice leads to hypoinsulinemia and type 2 diabetes symptoms of the terminal stage, along with leanness, hyperglycemia, and hyperlipidemia, under HFD feeding condition. Additionally, GPR84 deficiency partially attenuated the metabolic benefits provided by MCT. Our results may contribute to the understanding of the biological functions of MCFAs as signaling molecules.

## AUTHOR CONTRIBUTIONS

AN performed the experiments, interpreted data, and wrote the paper; RO‐K performed the experiments, interpreted data, and wrote the paper; YM performed the experiments and wrote the paper; HN performed the experiments and wrote the paper; MI interpreted data and wrote the paper; TI interpreted data and wrote the paper; IK supervised the project, interpreted data, and wrote the paper; IK had primary responsibility for the final content. All authors read and approved the final manuscript.

## DISCLOSURES

All authors declare no other competing interests.

## Supporting information


Data S1.

## Data Availability

All data generated or analyzed during this study are included in this published article and its Supplementary Material files or are available from the corresponding authors upon reasonable request. Sequence data for 16S rRNA gene has been deposited in DNA Data Bank of Japan (DDBJ) under accession No. DRA018990 and all of the data relevant to this study have been deposited in the Dryad database (Doi: 10.5061/dryad.c866t1gg4).
