# Supplementary material for: Medium‐chain fatty acid receptor GPR84 deficiency leads to metabolic homeostasis dysfunction in mice fed high‐fat diet
Source: FASEB Bioadv. 2024 Oct 17;6(11):526–38. doi: 10.1096/fba.2024-00075 (PMC11539033; doi:10.1096/fba.2024-00075)
Supplement: Supplementary file 1 — Data S1. [file FBA2-6-526-s001.docx]

**Medium-chain fatty acid receptor GPR84 deficiency leads to metabolic homeostasis dysfunction in mice fed high-fat diet**

## Akari Nishida, Ryuji Ohue-Kitano, Yuki Masujima, Hazuki Nonaka, Miki Igarashi, Takako Ikeda, Ikuo Kimura

# Supplementary Information

# Tables 1–2

**Supplementary Table 1**

#
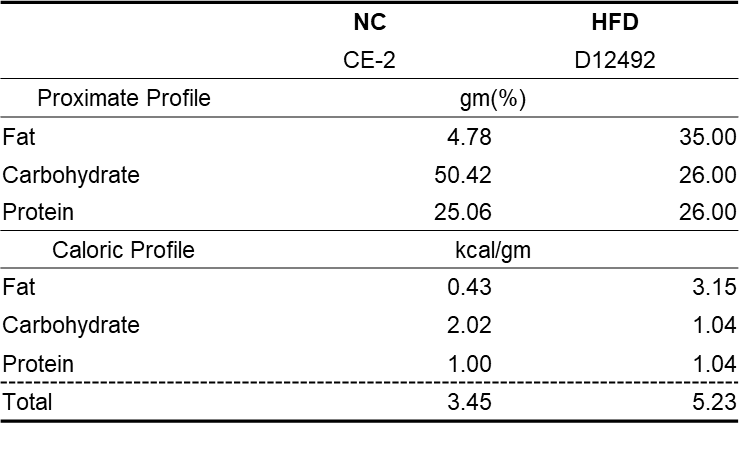


# Supplementary Table 1. Composition of normal chow and high-fat diet.

**Supplementary Table 2**


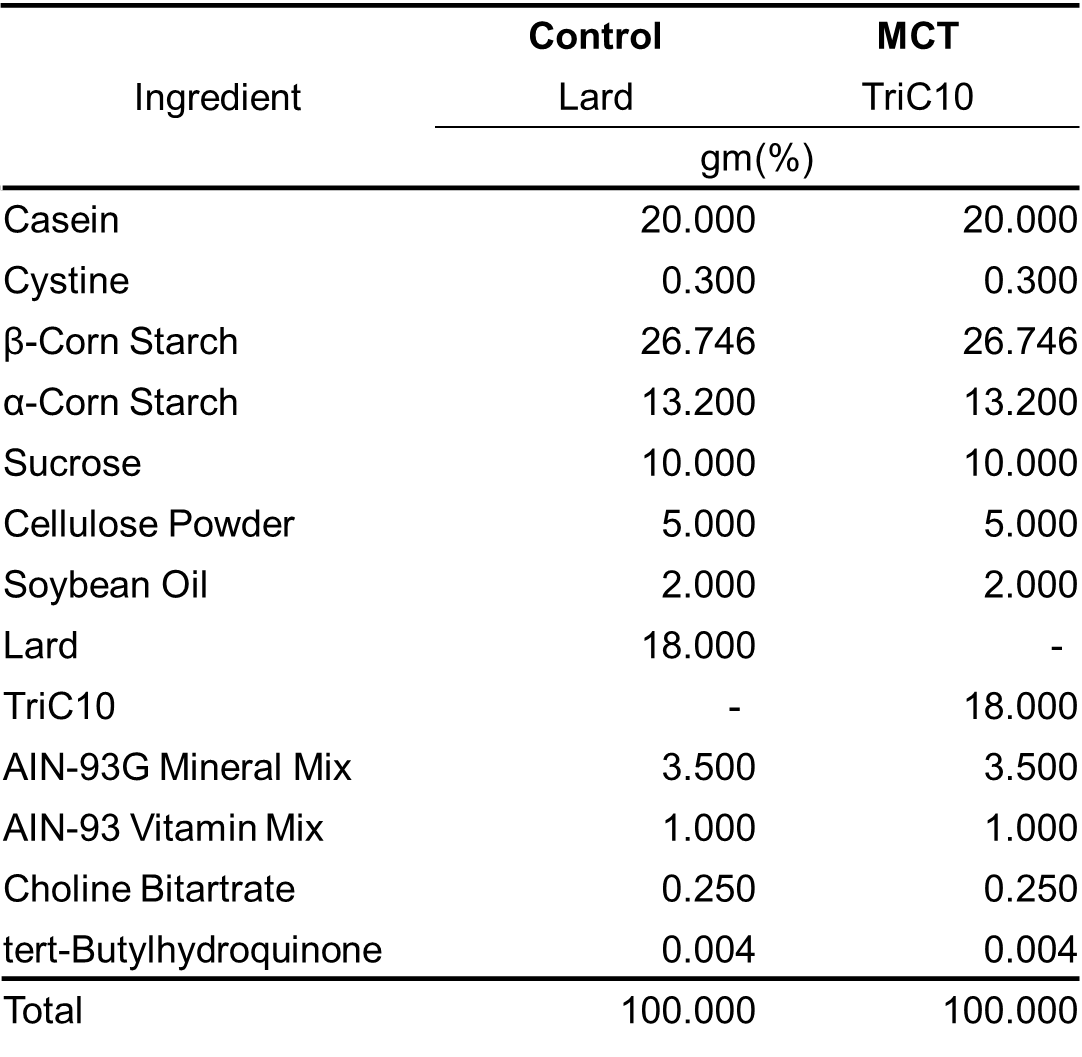


**Supplementary Table 2.** Dietary composition in the medium-chain triglyceride (MCT, TriC10) intake experiments. Lard, Lard diet; TriC10, decanoate (C10:0) triglyceride diet.
